# Cross-sectional association of physical activity levels with risks of sarcopenia among older Taiwanese adults

**DOI:** 10.1186/s12877-024-05087-x

**Published:** 2024-06-27

**Authors:** Chi-Hsuan Tsai, Yung Liao, Shao-Hsi Chang

**Affiliations:** 1 Graduate Institute of Sport, Leisure and Hospitality Management, National Taiwan Normal University, Taipei, Taiwan; 2 Department of Physical Education and Sport Sciences, National Taiwan Normal University, Taipei, Taiwan; 3 Faculty of Sport Sciences, Waseda University, Tokorozawa, Japan

**Keywords:** Physical activity recommendations, SARC-F, Exercise intensity

## Abstract

**Objectives:**

The updated World Health Organization 2020 guidelines strongly recommend an optimal physical activity level of 150–300 min/week for older adults. However, few studies have examined the relationship between different levels of physical activity and sarcopenia. Therefore, the purpose of this study was to investigate the cross-sectional associations between overall physical activity levels, gender, intensity, and the risk of sarcopenia among older Taiwanese adults.

**Methods:**

A nationwide cross-sectional telephone survey of older adults (≥ 65 years) was conducted in Taiwan from October 2019 to January 2020. Participants were interviewed to collect self-reported data on their level of physical activity (measured by the Taiwanese version of the IPAQ-SF), sarcopenia risk (measured by the SARC-F questionnaire), and sociodemographics.

**Results:**

A total of 1068 older adults were surveyed. Compared with the optimal physical activity level recommendations in the WHO guidelines and after adjusting for potential confounders and proposing an association independent of sedentary behavior, older adults with insufficient physical activity levels (< 150 min/week) were more likely to have a higher risk of sarcopenia (OR: 3.24; CI: 1.67–6.27), whereas older adults who exceeded physical activity guidelines (> 300 min/week) were more likely to have a lower risk of sarcopenia (OR: 0.39; CI: 0.20–0.78). Maintaining moderate-intensity physical activity is essential for older adults, as physical activity that exceeds the guidelines can significantly lower the risk of sarcopenia; meanwhile, insufficient physical activity can greatly increase it. Also, there seems to be a similar association between sarcopenia risk across different physical activity levels in vigorous-intensity physical activities in older adults. However, due to the small number of sarcopenia-risk participants who met or exceeded vigorous-intensity physical activity levels, further comparisons between different vigorous-intensity physical activity levels did not show significant differences in sarcopenia risk. Additionally, insufficient physical activity was found to be an important risk factor for sarcopenia in both genders, while physical activity that exceeded the guidelines prevented sarcopenia in females.

**Conclusions:**

The findings of this study highlight the potential dose-response relationship related to physical activity. The 2020 WHO guidelines provide the public with minimum recommendations for physical activity. However, exceeding these recommended levels appears to be more effective in preventing sarcopenia in older adults and may offer even greater health benefits. Future research should further explore whether exceeding these guidelines could result in additional health benefits.

## Introduction

The number and proportion of older adults in the population are rapidly increasing. In 2023, the global population aged 65 years or above constituted 10% of the total, and it is projected that this age group will represent 1 in every 6 people worldwide by 2050 [1, 2]. In Taiwan, the percentage of the older adult population rose to 18.37% in 2023, which is faster than the global aging rate, and the country is expected to become a rapidly aging society (i.e., a society with an older adult population of more than 20%) by 2025 [3]. With the advancement of medical resources and the improvement of public health, people’s life expectancy is extended [4]. From 1991 to 2022, the average life expectancy of Taiwanese people increased from 74.26 years to 79.84 years over 30 years [5]. With prolonged longevity, older adults may be more likely to suffer from skeletal muscle loss, reduced strength, or reduced physical performance as they age [6, 7].

Sarcopenia is a geriatric syndrome defined as a disorder characterized by loss of muscle mass, strength, and function (physical performance) [8, 9]. Older adults may be at high risk of sarcopenia as they age, likely due to factors such as inactivity, chronic diseases, anorexia, and nutritional imbalances [10]. Sarcopenia is characterized by feelings of weakness, difficulty with balance, and susceptibility to fractures. It is associated with consequences such as diminished physical ability, low quality of life, and increased mortality [8, 9]. The prevalence of sarcopenia in Taiwan in adults over 65 years old is approximately 3.9–7.3% [11, 12]. Sarcopenia-related research in Taiwan in the past has been inconsistent regarding the prevalence of the disease in relation to gender [12–16]. In most studies, males show a lower prevalence of sarcopenia than females [13], but in some studies, males exhibit a higher rate of sarcopenia than females [12, 13, 16]. It is certain that the aging population will increase each year, leading to a rise in sarcopenia [17]. To date, there is no specific drug treatment for sarcopenia [18]. However, through nonpharmaceutical interventions such as diet and physical activity, sarcopenia can be improved, disease progression can be reduced, and quality of life can be enhanced [19].

Physical activity (PA) is considered one of the most effective intervention strategies for reducing the risk of sarcopenia [20, 21]. Physical activity has been shown to lower the prevalence of sarcopenia in older adults, as confirmed by the results of a systematic review and meta-analysis [21]. According to previous studies, physical inactivity may result in sarcopenia [22–24]. Physical activity can increase muscle strength and muscle mass in older adults [25, 26], which has many beneficial effects on sarcopenia, including reduced apoptosis, reduced oxidative stress, anti-inflammation, improved insulin-glucose dynamics, enhanced quality and quantity of muscle proteins and mitochondria, skeletal muscle hypertrophy, positive neuromuscular adaptations, and enhanced muscle blood supply [27, 28]. Past research has indicated that the risk of sarcopenia varies with different intensities of physical activity. Older adults who engage in moderate to vigorous levels of physical activity are less likely to develop sarcopenia than those who engage in light physical activity [14, 29–31]. To promote physical activity, the World Health Organization {WHO} published guidelines on physical activity and sedentary behavior in 2020 that provide evidence-based public health recommendations [32, 33]. As part of this document, it is emphasis on the importance of regular physical activity. It strongly recommends that the physical activity level (PAL) for older adults should include engaging in at least 150–300 min of moderate-intensity physical activity, 75–150 min of vigorous-intensity physical activity, or a combination of moderate- and vigorous-intensity physical activity each week [32, 33]. Those guidelines also include physical activity time limits for physical activity in older adults and recommend health benefits within that limited range. However, the guidelines based on moderate evidence conditionally suggest that older adults may have additional health benefits beyond the recommended range of physical activity levels.

Although some meta-analyses have confirmed that physical activity in older adults can reduce the incidence of sarcopenia and that older adults with sarcopenia can improve their condition through physical activity [19, 21, 34], this study found three research gaps. Notably, only a few studies have included physical activity levels that exceed the guidelines as one of their categories, and few articles have discussed the relationship between physical activity levels and sarcopenia in older adults. Therefore, this study aims to determine the cross-sectional associations of overall physical activity levels, different genders, and intensities with the risk of sarcopenia among older Taiwanese adults.

## Methods

### Participants and procedures

This study employed a stratified random sampling method. Initially, the population of Taiwanese adults aged 65 or above, which totaled 3,607,127, was divided into four regions (East, South, West, and North). Subsequently, further grouping was conducted based on gender (male, female) and age (65–74 years, 75 years and above), forming multiple subgroups. Participants from each subgroup were randomly selected by dialing landline telephone numbers from the national household directory between October 2019 and January 2020. A telephone survey was conducted by a professional interview company as a cross-sectional study. The interviewers underwent at least 8 h of formal training before conducting interviews, and the interviews were based on structured and standardized questionnaires. During the interview process, the interviewer ensured the participant could understand and answer the questions logically. The question was suspended if the participant couldn’t understand or provide a logical answer. Incomplete investigations due to interruption or disconnection of the call process were excluded. In total, 2,352 older adults were interviewed, of which 1,068 completed the survey, resulting in a response rate of 45.4%. Before the telephone interview, each participant was given oral informed consent and thanked afterward. This study was reviewed and approved by the Research Ethics Committee of National Taiwan Normal University (REC number: 201706HM020).

### Measures

#### Physical activity

Physical activity was measured using the Taiwanese version of the International Physical Activity Questionnaire-Short Form (IPAQ-SF) [35] which has been recommended as a cost-effective method for assessing physical activity and has also been used in numerous studies. Its test-retest reliability (*r* = .78) and criterion validity (*r* = .31-0.41) were both high and acceptable [36]. The IPAQ-SF comprises 7 items that assess the frequency and duration of vigorous-intensity activity, moderate-intensity activity, walking (classified as a moderate-intensity activity), and sitting behavior that participants have undertaken in the past week. The overall physical activity level for an individual is determined by combining minutes of walking and moderate-to-vigorous intensity activity. This questionnaire classifies physical activities into moderate-intensity and vigorous-intensity activities. In our study, following the WHO guidelines on physical activity and sedentary behavior recommended physical activity criteria [32, 33]. We categorized physical activity into three levels: insufficient physical activity level (< 150 min/week, not achieving the physical activity recommendation), sufficient physical activity level (≥ 150–300 min/week, meeting the physical activity recommendation), and physical activity level exceeding guidelines (> 300 min/week, beyond the physical activity recommendation). Additionally, this study independently examined the association between sarcopenia and physical activity levels among different genders and various intensities.

#### Sarcopenia

The SARC-F questionnaire [37], a screening tool, was rapidly implemented to identify individuals with possible sarcopenia. It has been proven to exhibit good internal consistency reliability and factorial, criterion, and construct validity for detecting people at risk for sarcopenia across multiple studies [38, 39]. The SARC-F screened for signs of sarcopenia through self-report, including five items: lack of strength, walking, rising from a chair, climbing stairs, and falling. Each item was scored on a 0–2 scale from easy to difficult [37]. The scores for all items were summed, and an individual with a SARC-F score ≥ 4 was classified as at risk of sarcopenia [37].

#### Covariates

Covariates for this study were demographic data collected through self-report standardized questionnaires, including gender (male or female), age (65–74 years or older 75 years), geographic region of residence (urban area or suburban area), education level (lower than university, university or higher), employment status (full-time or part-time), marital status (married or unmarried), living status (alone or with others), health behavior (i.e., smoking status (smoker or nonsmoker), alcohol consumption (drinker or nondrinker), diet (balanced or unbalanced)), height and weight. Furthermore, the body mass index (BMI) was calculated by dividing the recorded weight (kg) by the square of the height (${\text{m}}^{\text{2}}$). According to BMI standards provided by the Ministry of Health and Welfare of Taiwan, the participants were divided into underweight (BMI < 18.5 kg/ m^2^) and normal weight (18.5 kg/ m^2^ ≤ BMI < 24 kg/m^2^), and overweight (BMI ≥ 24 kg/${\text{m}}^{\text{2}}$).

### Statistical analysis

The statistical analysis of this study used IBM SPSS Statistics for Windows, version 24.0 (IBM Corporation., Armonk, NY, USA), and the significance level was set at *p* < .05. Chi-square tests were used to compare differences in demographic characteristics and physical activity levels between the two groups at risk of sarcopenia. In addition, three models were established using binary logistic regression models to predict odds ratios (ORs) for different levels of physical activity and sarcopenia risk in older adults. This analysis included 95% confidence intervals (CIs) before and after adjustment for covariates: Model 1 was unadjusted; Model 2 was adjusted for age and gender variables; and Model 3 was adjusted for all potential confounders (i.e., age, gender, education level, employment status, marital status, living status, smoking status, alcohol consumption, diet, BMI) and proposed an association independent of sedentary behavior. Furthermore, this study independently investigated the association between sarcopenia and physical activity levels among different genders and at various intensities.

## Results

### Participant characteristics

Table 1 shows sociodemographic variables and health behavior in the total sample and separately by sarcopenia risk. A total of 1068 participants (47.3% male; aged 72.2 (± 5.7) years) were included in this analysis. Among them, 7.3% of participants were identified as being at risk of sarcopenia. Most of the participants lived in an urban area (64.0%), had a lower than university education level (74.7%), worked part-time (88.3%), married (77.6%), and lived with others (90.7%). A significant proportion of participants were non-smokers (92.8%), abstained from alcohol (90.1%), adhered to a healthy diet (78.7%), had a body mass index (BMI) less than 24 kg/m^2^, indicating underweight or normal weight (52.9%), and reported under 9 h per day of sedentary behavior (94.5%). Participants predominantly engaged in physical activity levels that exceeded the guidelines (> 300 min/week) (67%). According to the physical activity levels divided by intensity, participants were more active (> 300 min/week) in moderate-intensity physical activities (including walking time) (65.3%) but less active (< 75 min/week) in vigorous-intensity physical activities (84.3%). We used a significance level of *p* < .05 in the chi-square test for these categorical variables, which showed that gender, age, education level, employment status, marital status, living status, health behaviors (smoking status, alcohol consumption, and healthy diet) were associated with sarcopenia. Therefore, we included these covariates and proposed an association independent of sedentary behavior as adjustment factors in the following analysis.

**Table 1:** Characteristics of the study participants (*N* = 1068)

	Total (*N* = 1068)			Sarcopenia				*p*-value
				Normal (*n* = 990) 92.7%		At risk (*n* = 78) 7.3%		
	*n*	%		*n*	%	*n*	%	
**Gender**								**.00** ^a*^
Male	505		47.3%	484	95.8%	21	4.2%	
Female	**563**		**52.7%**	506	89.9%	57	10.1%	
**Age**								**.00** ^a*^
Mean (± SD)	72.2 (± 5.7)			71.9(± 5.5)		75.4(± 6.7)		**.00** ^b*^
65–74 years	**731**		**68.4%**	693	94.8%	38	5.2%	
75 + years	337		31.6%	297	88.1%	40	11.9%	
**Region of residence**								.81^a^
Urban area	**684**		**64.0%**	635	92.8%	49	7.2%	
Suburban area	384		36.0%	355	92.4%	29	7.6%	
**Education level**								**.02** ^a*^
Lower than university	**798**		**74.7%**	731	91.6%	67	8.4%	
University or higher	270		25.3%	259	95.9%	11	4.1%	
**Employment status**								**.00** ^a*^
Full-time	125		11.7%	124	99.2%	1	0.8%	
Part-time	**943**		**88.3%**	866	91.8%	77	8.2%	
**Marital status**								**.00** ^a*^
Married	**829**		**77.6%**	781	94.2%	48	5.8%	
Unmarried	239		22.4%	209	87.4%	30	12.6%	
**Living status**								**.02** ^a*^
Alone	99		9.3%	86	86.9%	13	13.1%	
With others	**969**		**90.7%**	904	93.3%	65	6.7%	
**Smoking status**								**.04** ^a*^
No	**991**		**92.8%**	914	92.2%	77	7.8%	
Yes	77		7.2%	76	98.7%	1	1.3%	
**Alcohol consumption**								**.01** ^a*^
No	**962**		**90.1%**	885	92%	77	7.8%	
Yes	106		9.9%	105	99.1%	1	0.9%	
**Healthy diet**								**.00** ^a*^
No	227		21.3%	199	87.7%	28	12.3%	
Yes	**841**		**78.7%**	791	94.1%	50	5.9%	
**BMI (kg/** ${\text{m}}^{\text{2}})$								.32^a^
Mean (± SD)	24.1 (± 3.5)			24.0(± 3.4)		24.9(± 4.5)		**.03** ^b*^
Underweight & Normal weight (BMI < 24 kg/m^2^)	**565**		**52.9%**	528	93.5%	37	6.5%	
Overweight (BMI ≥ 24 kg/m^2^)	503		47.1%	462	91.8%	41	8.2%	
**Sedentary behavior**								**.02** ^a*^
Mean (± SD)	271.8(± 136.6)			269.6(± 134.6)		299.7(± 158)		**.02** ^b*^
< 9 h/dairy	1009		94.5%	940	93.2%	69	6.8%	
≥ 9 h/dairy	59		5.5%	50	84.7%	9	15.3%	
**Overall physical activity levels**								**.00** ^a*^
Mean (± SD)	772.9 (± 797.2)			809.9(± 804.3)		303.3(± 506.7)		**.00** ^b*^
Insufficient PAL (< 150 min/week)	168		15.7%	129	76.8%	39	23.2%	
Sufficient PAL (≥ 150–300 min/week)	184		17.3%	167	90.8%	17	9.2%	
Exceeded PAL (> 300 min/week)	**716**		**67%**	694	96.9%	22	3.1%	
**Moderate-intensity PALs (including walking)**								**.00** ^a*^
Mean (± SD)	701.6 (± 723.9)			733.6(± 729.2)		295.5(± 501.6)		**.00** ^b*^
Insufficient PAL (< 150 min/week)	188		17.6%	148	78.7%	40	21.3%	
Sufficient PAL (≥ 150–300 min/week)	183		17.1%	166	90.7%	17	9.3%	
Exceeded PAL (> 300 min/week)	**697**		**65.3%**	676	97%	21	3%	
**Vigorous-intensity PALs**								**.01** ^a*^
Mean (± SD)	71.2 (± 243.4)			76.2(± 251.9)		7.82(± 37.8)		**.00** ^b*^
Insufficient PAL (< 75 min/week)	**900**		**84.3%**	825	91.7%	75	8.3%	
Sufficient PAL (≥ 75–150 min/week)	42		3.9%	41	97.6%	1	2.4%	
Exceeded PAL (> 150 min/week)	126		11.8%	124	98.4%	2	1.6%	

**Abbreviations**: BMI: body mass index; PAL: physical activity level

${ }^{a*}$*P* < .05 indicated a significant difference for categorical variables compared with χ^2^ tests

${ }^{b*}$*P* < .05 indicated a significant difference for continuous variables compared with *t-tests*

### Overall physical activity levels with risks of Sarcopenia among older adults

Table 2 depicts the association between overall physical activity levels and the risk of sarcopenia among older Taiwanese adults using binary logistic regression models.

In Model 1 (unadjusted model), both inactivity (< 150 min/week) and physical activity levels exceeding the guidelines (> 300 min/week) in older adults were significantly associated with sarcopenia risk. As a result, older adults who did not achieve the recommended weekly amount of physical activity might have a 197% increased risk of sarcopenia (OR: 2.97; 95% CI: 1.61–5.49; *p* < .01); in contrast, older adults who are beyond the recommended amount of physical activity might have a 69% lower risk of sarcopenia (OR: 0.31; 95% CI: 0.16–0.60; *p* < .01).

In Model 2, age and gender variables were added for binary regression analysis. Both inactivity and physical activity levels exceeding the guidelines were significantly associated with sarcopenia risk. Excluding age and gender, older adults who do not achieve the recommended level of weekly physical activity might have a 202% increased risk of sarcopenia (OR: 3.02; 95% CI: 1.61–5.65; *p* < .01); conversely, those who exceed the recommended amount of physical activity might have a 66% lower risk of sarcopenia (OR: 0.34; 95% CI: 0.18–0.66; *p* < .01).

In Model 3, all relevant covariates and sedentary behavior were included in the analysis. Inactivity and physical activity levels exceeding the guidelines in older adults were also associated with sarcopenia risk. The findings indicated that older adults who do not meet the recommended weekly physical activity might have a 224% increased risk of sarcopenia (OR: 3.24; 95% CI: 1.67–6.27; *p* < .01). On the other hand, older adults who exceed the recommended amount of physical activity might have a 61% decreased risk of sarcopenia (OR: 0.39; 95% CI: 0.20–0.78; *p* < .01).

**Table 2:** Physical activity levels with risks of sarcopenia among older Taiwanese adults

		Physical activity levels		
		Insufficient PAL (< 150 min/week)	Sufficient PAL (≥ 150–300 min/week)	Exceeded PAL (> 300 min/week)
**Model 1** ${ }^{a}$	OR	2.97	1.00	0.31
	95% CI	(1.61, 5.49)	-	(0.16, 0.60)
	*p-value*	0.001**	-	0.000**
**Model 2** ${ }^{b}$	OR	3.02	1.00	0.34
	95% CI	(1.61, 5.65)	-	(0.18, 0.66)
	*p-value*	0.001**	-	0.001**
**Model 3** ${ }^{c}$	OR	3.24	1.00	0.39
	95% CI	(1.67, 6.27)	-	(0.20, 0.78)
	*p-value*	0.001**	-	0.007**

**Abbreviations**: PAL: physical activity level; OR: odds ratio; CI: confidence interval

$${ }^{a}$$Model 1: unadjusted

${ }^{b}$Model 2: adjusted for age and gender

${ }^{c}$Model 3: adjusted for age, gender, education level, employment status, marital status, living status, smoking status, alcohol consumption, diet, and sedentary behavior

* *p* < .05

** *p* < .01

***p < .001

### Intensities of physical activity levels with risks of Sarcopenia among older adults

Table 3 uses binary logistic regression models to explore further the association between different intensities of physical activity levels and the risk of sarcopenia among older adults in Taiwan.

In Model 1 (unadjusted), both insufficient (< 150 min/week) and physical activity levels exceeding the guidelines (> 300 min/week) of moderate-intensity activity were significantly associated with the risk of sarcopenia. In contrast, vigorous-intensity physical activity did not correlate statistically with sarcopenia risk. Therefore, older adults not meeting the recommended weekly moderate physical activity level (< 150 min/week) might have a 164% increased risk of sarcopenia (OR: 2.64; 95% CI: 1.44–4.85; *p* < .01). Conversely, those exceeding the recommended level of moderate physical activity (> 300 min/week) might lower their risk of sarcopenia by 70% (OR: 0.30; 95% CI: 0.16–0.59; *p* < .001).

In Model 2, after adjusting for age and gender, insufficient moderate physical activity level (< 150 min/week) and moderate physical activity level exceeding the guidelines (> 300 min/week) were also significantly associated with the risk of sarcopenia. Vigorous physical activity did not reach statistical significance, indicating no significant relationship with the risk of sarcopenia. As a result, the risk of sarcopenia might have a 178% increase in older adults who do not meet the recommended physical activity level per week (< 150 min/week) (OR: 2.78; 95% CI: 1.49–5.18; *p* < .01). In contrast, older adults exceeding the recommended level of moderate physical activity (> 300 min/week) per week might have a 67% lower risk of sarcopenia (OR: 0.33; 95% CI: 0.17–0.65; *p* < .01).

In Model 3, after adjusting for all relevant covariates and independently controlling for sedentary behavior, the results indicated that both insufficient moderate physical activity level (< 150 min/week) and moderate physical activity level exceeding the guidelines (> 300 min/week) remained significantly associated with the risk of sarcopenia. Vigorous physical activity did not reach statistical significance, indicating no significant relationship with the risk of sarcopenia. Thus, older adults who do not achieve the recommended level of weekly physical activity might have a 201% increased risk of sarcopenia (OR: 3.01; 95% CI: 1.56–5.77; *p* < .01). On the other hand, those who exceed the recommended amount of physical activity might have a 62% lower risk of sarcopenia (OR: 0.38; 95% CI: 0.19–0.75; *p* < .01).

**Table 3:** Intensities of PALs with risks of sarcopenia among older Taiwanese adults

Moderate-intensity PALs (including walking)				
		Insufficient PAL (< 150 min/week)	Sufficient PAL (≥ 150–300 min/week)	Exceeded PAL (> 300 min/week)
**Model 1** ${ }^{a}$	OR	2.64	1.00	0.30
	95% CI	(1.44, 4.85)	-	(0.16, 0.59)
	*p-value*	0.002**	-	0.000***
**Model 2** ${ }^{b}$	OR	2.78	1.00	0.33
	95% CI	(1.49, 5.18)	-	(0.17, 0.65)
	*p-value*	0.001**	-	0.001**
**Model 3** ${ }^{c}$	OR	3.01	1.00	0.38
	95% CI	(1.56, 5.77)	-	(0.19, 0.75)
	*p-value*	0.001**	-	0.005**
**Vigorous intensity PALs**				
		Insufficient PAL (< 75 min/week)	Sufficient PAL (≥ 75–150 min/week)	Exceeded PAL (> 150 min/week)
**Model 1** ${ }^{a}$	OR	3.73	1.00	0.66
	95% CI	(0.51, 27.48)	-	(0.06, 7.48)
	*p-value*	0.197	-	0.738
**Model 2** ${ }^{b}$	OR	3.18	1.00	0.70
	95% CI	(0.43, 23.71)	-	(0.06, 7.99)
	*p-value*	0.259	-	0.773
**Model 3** ${ }^{c}$	OR	2.01	1.00	0.59
	95% CI	(0.26, 15.23)	-	(0.05, 6.82)
	*p-value*	0.501	-	0.669

**Abbreviations**: PAL: physical activity level; OR: odds ratio; CI: confidence interval

$${ }^{a}$$Model 1: unadjusted

${ }^{b}$Model 2: adjusted for age and gender

${ }^{c}$Model 3: adjusted for age, gender, education level, employment status, marital status, living status, smoking status, alcohol consumption, diet, and sedentary behavior

* *p* < .05

** *p* < .01

***p < .001

### Across genders of overall physical activity levels with risks of Sarcopenia among older adults

Table 4 applies binary logistic regression analysis to examine the relationship between overall physical activity levels and the risk of sarcopenia among older Taiwanese adults of different genders.

In Model 1 (unadjusted), both older males and females showed that insufficient overall physical activity (< 150 min/week) was significantly associated with an increased risk of sarcopenia. Older males not meeting the recommended weekly physical activity levels might face an 800% increased risk of sarcopenia (OR: 9.00; 95% CI: 1.97–41.06; *p* < .01). In contrast, insufficient overall physical activity (< 150 min/week) in older females might add 117% to the risk of sarcopenia (OR: 2.17; 95% CI: 1.06–4.44; *p* < .05). Furthermore, physical activity levels exceeding the guidelines (> 300 min/week) in older males did not reach statistical significance, suggesting no significant association between exceeding the guidelines and the risk of sarcopenia in older males. However, unlike males, physical activity levels exceeding the guidelines (> 300 min/week) in older females were significantly associated with the risk of sarcopenia. Older females who exceed the recommended weekly physical activity levels might have a 72% lower risk of developing sarcopenia (OR: 0.28; 95% CI: 0.13–0.57; *p* < .001).

In Model 2 (adjusted for age), insufficient overall physical activity (< 150 min/week) continued to be significantly associated with an increased risk of sarcopenia in both older males and females. Older males not meeting the recommended weekly physical activity levels might face an 862% increase in sarcopenia risk (OR: 9.62; 95% CI: 2.08–44.52; *p* < .01), while older females not meeting these levels could see a 109% increase in risk (OR: 2.09; 95% CI: 1.02–4.30; *p* < .05). Meanwhile, physical activity exceeding the guidelines (> 300 min/week) in older males did not show statistical significance, suggesting no significant association between such high levels of physical activity and sarcopenia risk. However, for older females, physical activity levels exceeding the guidelines were significantly associated with a reduced risk of sarcopenia. Older females who surpass the recommended weekly physical activity levels might have a 71% decrease in risk of developing sarcopenia (OR: 0.29; 95% CI: 0.14–0.61; *p* < .001).

In Model 3 (adjusted for all covariates and sedentary behavior), insufficient overall physical activity (< 150 min/week) in older males remained significantly associated with an increased risk of sarcopenia. The risk of developing sarcopenia for older males not meeting the recommended weekly physical activity might rise to 1152% (OR: 12.52; 95% CI: 2.46–63.68; *p* < .01). However, for older females, not reaching the recommended weekly physical activity level (< 150 min/week) could increase the risk of sarcopenia by 99%, although this result was not statistically significant, with the *p*-value nearing the threshold of significance (OR: 1.99; 95% CI: 0.92–4.32; *p* = .081). On the other hand, physical activity exceeding the guidelines (> 300 min/week) still showed no significant association with the risk of sarcopenia in older males. In older females, conversely, physical activity levels that exceed the guidelines (> 300 min/week) were significantly linked to a 67% reduction in sarcopenia risk (OR: 0.33; 95% CI: 0.16–0.71; *p* < .01).

**Table 4:** Across genders of overall PALs with risks of sarcopenia among older adults

Physical Activity Level of Older Males				
		Insufficient PAL (< 150 min/week)	Sufficient PAL (≥ 150–300 min/week)	Exceeded PAL (> 300 min/week)
**Model 1** ${ }^{a}$	OR	9.00	1.00	0.60
	95% CI	(1.97, 41.06)	-	(0.11, 3.13)
	*p-value*	0.005**	-	0.540
**Model 2** ${ }^{b}$	OR	9.62	1.00	0.67
	95% CI	(2.08, 44.52)	-	(0.13, 3.57)
	*p-value*	0.004**	-	0.642
**Model 3** ${ }^{c}$	OR	12.52	1.00	0.79
	95% CI	(2.46, 63.68)	-	(0.14, 4.43)
	*p-value*	0.002**	-	0.792
**Physical Activity Level of Older Females**				
		Insufficient PAL (< 150 min/week)	Sufficient PAL (≥ 150–300 min/week)	Exceeded PAL (> 300 min/week)
**Model 1** ${ }^{a}$	OR	2.17	1.00	0.28
	95% CI	(1.06, 4.44)	-	(0.13, 0.57)
	*p-value*	0.034*	-	0.001**
**Model 2** ${ }^{b}$	OR	2.09	1.00	0.29
	95% CI	(1.02, 4.30)	-	(0.14, 0.61)
	*p-value*	0.045*	-	0.001**
**Model 3** ${ }^{c}$	OR	1.99	1.00	0.33
	95% CI	(0.92, 4.32)	-	(0.16, 0.71)
	*p-value*	0.081	-	0.004**

**Abbreviations**: PAL: physical activity level; OR: odds ratio; CI: confidence interval

$${ }^{a}$$Model 1: unadjusted

${ }^{b}$Model 2: adjusted for age

${ }^{c}$Model 3: adjusted for age, education level, employment status, marital status, living status, smoking status, alcohol consumption, diet, and sedentary behavior

* *p* < .05

** *p* < .01

***p < .001

## Discussions

In Taiwan, this is the first national study to investigate the association between physical activity levels and the risk of sarcopenia in older adults, as recommended by the WHO 2020 guidelines on physical activity and sedentary behavior. The main finding is that physical activity levels exceeding the guidelines appear to be more effective in preventing sarcopenia, especially in the case of older adults engaging in a large number of moderate-intensity activities or for females. An insufficient physical activity level, on the other hand, may lead to an increased risk of sarcopenia, particularly in the case of inadequate moderate activities or for males. These unique findings are informative for policymakers and intervention designers.

This study has four critical implications.

Firstly, in terms of participant characteristics, this study conducted a rapid screening of muscle function using the SARC-F questionnaire, which is recommended by both the European Working Group on Sarcopenia in Older People (EWGSOP) and the Asian Working Group for Sarcopenia (AWGS) [7, 9]. The results identified that the proportion of participants at risk of sarcopenia (score ≥ 4) was 7.3%. According to a 2017 National Health Interview Survey in Taiwan, 7.0% of 2,163 community-dwelling residents aged 65 and above were also likely to have sarcopenia using the SARC-F [40]. Both rates of risk sarcopenia fall within the range reported in previous literature for the prevalence of sarcopenia among Older Taiwanese Adults (3.9–7.3%) [11, 12]. Additionally, there are differences in overall physical activity levels between populations with and without the risk of sarcopenia. Residual analysis showed that those at risk of sarcopenia had lower physical activity levels compared to those without risk, aligning with the findings of a five-year longitudinal study [41], which indicated that older adults with higher activity levels have a significantly lower incidence of sarcopenia. The noteworthy aspect of this study is that the overall physical activity level of older adults in Taiwan is quite active, with 68.3% exceeding the WHO’s recommended physical activity levels per week. Compared to other countries, the study indicates that 15.7% of Taiwanese adults over 65 are physically inactive, which is less than the inactivity prevalence of 31.2% in American and 50% in Australian older adults [42, 43].results show that the risk of sarcopenia is significantly higher in females than in males.

Secondly, the results of overall physical activity levels with risks of sarcopenia among older adults. The results indicate that insufficient physical activity may increase the risk of developing sarcopenia, consistent with previous literature reviews [21–24]. Conversely, physical activity is beneficial in lowering the prevalence of sarcopenia in older adults [21, 25, 34, 44]. This finding aligns with the previous systematic review and meta-analysis study [21], confirming that physical inactivity is a significant risk factor for sarcopenia in older adults and that increasing physical activity levels effectively protects muscle mass and prevents sarcopenia. Another key finding of this study is that physical activity levels exceeding the guidelines might effectively reduce the risk of sarcopenia. This may be related to the fact that physical activity can increase muscle strength and muscle mass, and improve physiological function in older adults [25, 27, 28, 34]. Although the WHO increased activity level recommendations in 2020, engaging in even higher levels of physical activity may further reduce the risk of sarcopenia. Few studies have explored physical activity levels exceeding these guidelines in the past. According to a cross-sectional study [14], higher levels of physical activity are associated with approximately half the odds ratio for sarcopenia, highlighting physical activity as a key factor in reducing the risk of sarcopenia. While the World Health Organization strongly recommends maintaining physical activity levels within a range that is beneficial for health, this study supports the moderate evidence mentioned by WHO that activity levels beyond the guidelines could provide additional health benefits for older adults [32, 33].

Thirdly, the study investigates the relationship between different intensities of physical activity and sarcopenia. The findings indicate that moderate-intensity physical activity has a similar association with the risk of sarcopenia as overall activity levels. However, this study finds no significant association between vigorous physical activity and an increased or decreased risk of sarcopenia. This lack of statistical difference is likely due to the small number of participants at risk of sarcopenia who engaged in vigorous physical activities at levels sufficient to meet or exceed the recommended guidelines. Only one out of the 43 participants who met the guidelines was at risk of sarcopenia, resulting in a larger confidence interval for this group. Specifically, older adults engaging in less than 150 min per week of moderate-intensity activity may significantly increase the risk of sarcopenia. Conversely, exceeding 300 min per week of such activities may reduce the risk of developing sarcopenia by 62%. These findings align with previous research [29–31]. A previous study conducted a three-month intervention in older females, demonstrating that moderate-intensity exercise effectively improves body composition, induces physical changes, and reduces oxidative damage [29]. This plays a crucial role in preventing or treating muscle loss in older adults. In a five-year longitudinal study [31], which objectively measured the physical activity of older Japanese adults using accelerometers, it was found that those walking at least 7,000 to 8,000 steps per day or engaging in at least 15 to 20 min of daily exercise at an intensity greater than 3 METs significantly lowered their risk of muscle loss. This highlights the importance of moderate-intensity activities for older adults.

Finally, this study conducted a sensitivity analysis based on gender. Regarding the gender-specific risks for sarcopenia, it was found that insufficient physical activity is closely related to an increased risk of sarcopenia in older Taiwanese males, while it is less associated with older females. However, physical activity levels exceeding the guidelines in older males did not show a significant correlation with the risk of sarcopenia; conversely, for females, physical activity levels that exceed the guidelines significantly reduced the risk. These results suggest that the relationship between physical activity levels and the risk of sarcopenia may vary by gender. Insufficient physical activity is a significant risk factor for sarcopenia in older adults of both genders, but physical activity levels exceeding the guidelines may significantly prevent sarcopenia in females. These findings align with the previous systematic review and meta-analysis study [21], which found that physical activity significantly reduces the odds of suffering from sarcopenia, with a more substantial overall effect size in males (*Z* = 6.50) compared to females (*Z* = 3.79).

There were several limitations to this study. First, this study used a cross-sectional design and was unable to establish a causal relationship between physical activity and the risk of sarcopenia, establishing only an association. Despite this, such a design is appropriate for encompassing a wide range of older adults within a short period, thereby allowing an initial exploration of the association between the two. Secondly, the study uses self-reported questionnaires and telephone surveys to measure participants’ behavior and the risk of sarcopenia, which could have resulted in recall bias or the potential risk of social expectation bias; however, the IPAQ-SF and SARC-F questionnaires have been shown to be practical and applicable for extensive national epidemiological surveys. Thirdly, the main aspect of this study was to explore overall physical activity levels by calculating the total time spent walking and engaging in moderate to vigorous intensity activities. It categorized these activities according to the WHO’s recommended thresholds for moderate intensity (150–300 min/week) for older adults, which may have underestimated the results for vigorous intensity. However, this study also explored physical activities at both moderate and vigorous intensities separately. Furthermore, this study did not investigate the types of exercises (such as resistance training, aerobic training, etc.) or chronic diseases (such as stroke, diabetes, heart disease, etc.) among older adults, nor did it analyze the association between sedentary behavior and sarcopenia independently. Despite these limitations, this study still provides preliminary insights into physical activity levels exceeding the guidelines in older adults. It suggests possible ways to prevent or decrease the risk of sarcopenia in the future.

The WHO’s global status report on physical activity in 2022 continues to highlight the recommended range of physical activity [45]. Previous related reports have mainly focused on exploring the reduction of physical inactivity, confirming through numerous studies the importance of physical activity in reducing chronic diseases (such as hypertension, diabetes, and heart disease) and proposing related individual physical activity guidelines. In fact, the physical activity levels in many countries have already exceeded the guidelines. However, there is a lack of related research and consistent evidence demonstrating its feasibility. Furthermore, with the aging population trend and the increasing risk of sarcopenia, this issue should also be given attention, and specific physical activity guidelines should be further developed. Therefore, researchers in the future could attempt to use objective scientific measurement methods, such as accelerometers, to accurately analyze the different intensities of physical activity and sedentary behavior in older adults. Additionally, they could further incorporate more potential factors, such as exercise habits, types of exercise, chronic diseases, specific nutrient consumption, muscle mass, muscle strength, and muscle function, to develop a unified assessment method and strategies to prevent and alleviate for sarcopenia in older adults.

## Conclusions

This study highlights that even when physical activity levels exceed the recommendations of the WHO 2020 guidelines, they are more effective in preventing sarcopenia in older adults. Therefore, we should remind the public that the WHO’s physical activity recommendations are minimum guidelines, and exceeding these levels may be even more beneficial. This suggests a dose-response relationship, where increased physical activity leads to greater health benefits, potentially reducing the risk of sarcopenia by more than half. Future research require further cohort studies or even randomized controlled trials to confirm our findings.

## Data Availability

The datasets used and/or analyzed during the current study are available from the corresponding author upon reasonable request.

## Abbreviations

WHO: World Health Organization
ASWGS: Asian Sarcopenia Working Group
EWGSOP2: European Working Group on Sarcopenia in Older People
PA: physical activity
PAL: physical activity level
BMI: body mass index
IPAQ-SF: International Physical Activity Questionnaire-Short Form
SARC-F: questionnaire for sarcopenia (strength, assistance in walking, rising from a chair, climbing stairs, and falls)
OR: odds ratio; CI: confidence interval
